# How Can Robotic Devices Help Clinicians Determine the Treatment Dose for Post-Stroke Arm Paresis?

**DOI:** 10.3390/s25051612

**Published:** 2025-03-06

**Authors:** Ophélie Pila, Christophe Duret

**Affiliations:** Centre de Rééducation Fonctionnelle les Trois Soleils, Médecine Physique et de Réadaptation, Unité de Neurorééducation, 77310 Boissise-le-Roi, France; c.duret@les-trois-soleils.fr

**Keywords:** stroke, upper limb, dose, kinematic, recovery, stratification, robotic, difficulty

## Abstract

Upper limb training dose after stroke is usually quantified by time and repetitions. This study analyzed upper limb motor training dose in stroke participants (N = 36) using a more comprehensive approach. Participants, classified by initial motor severity (severe/moderate/mild) and recovery trajectory (good/poor), received daily robotic and occupational therapy. Treatment dose was reported using a multidimensional framework. Fugl-Meyer Assessment (FMA) score and robot-derived kinematic parameters (reach distance (cm), velocity (cm/s), accuracy (cm) and smoothness (number of velocity peaks)) were analyzed pre- and post-intervention. FMA scores (mean (SD)) improved significantly post-intervention in severe (+11 (12) pts; *p* < 0.001) and moderate (+13 (6) pts; *p* ≤ 0.01) impairment groups. In the severe group, good recoverers showed greater improvement (+18 (12) pts) than poor recoverers (+4 (4) pts). Despite similar robotic therapy duration (34 min/session) and number of movements (600–900/session) between good and poor recoverers, both groups experienced very different therapeutic plans in the use of physical modalities: good recoverers gradually moved from assisted to the unassisted then resisted modality. Kinematic analysis showed distinct patterns of motor improvement across severity levels, ranging from quantitative (reach distance/velocity) to qualitative (accuracy/smoothness) changes. This approach provides a more accurate description of the therapeutic dose by characterizing the movements actually performed and can help personalize rehabilitation strategies.

## 1. Introduction

Stroke remains a leading cause of disability in adults, with hemiparesis of the upper limb being the most common motor disorder [1]. Almost 2/3 of people after stroke have an unfavorable natural prognosis. Motor loss often persists in the chronic phase of stroke, limiting daily activities. Although significant advances have been made in understanding recovery mechanisms, optimizing rehabilitation strategies remains challenging.

The severity of the initial motor impairment is a major determinant of functional outcome. Studies have shown that the initial severity of the motor deficit is an important predictor of long-term motor outcomes [2]. Upper limb motor recovery is relatively predictable in people with mild impairment, but the patterns of motor recovery are highly variable in those with moderate to severe initial impairment [3]. Motor rehabilitation is based on activity-based training and aims to reduce motor deficits and improve functional abilities through learning- and use-dependent mechanisms [4], making it a crucial process for improving individuals’ quality of life.

Although the principles of motor learning have been clearly established (repetition, difficulty and goal-directed task exercises [5,6,7,8]), the required amount of training to stimulate brain plasticity is unclear. Current doses of motor retraining are not sufficient to exceed spontaneous recovery trajectories. Although motor rehabilitation is essential, the relationship between treatment dose and motor recovery is not yet fully understood. Understanding this relationship is particularly important for people with severe motor deficits, where the recovery profile seems less predictable than that of people with mild or moderate impairment [3].

Robotic motor rehabilitation offers repetitive, reproducible and quantifiable training for the paretic limb. The device records the content of training sessions in real-time so that at the end of each session [9,10,11,12] precise data are available on the number of repetitions, the actual time spent in the activity and the type of effort made (i.e., modality used, from assistance to resistance [13]).

Doses provided in post-stroke motor rehabilitation are both a crucial issue and a challenge facing clinicians and researchers alike. Indeed, treatment dose is a complex concept in which the quantity of active ingredients, their frequency and duration must be taken into consideration to achieve the best outcome. Hayward et al. [14] conceptualized a novel framework to provide an accurate description of dose, taking into account its multidimensional nature. This framework enables precise description, implementation, monitoring and reporting of the dose administered as part of a non-pharmacological intervention.

To date, studies quantifying the treatment administered to people after stroke have not described dose in relation to the severity of the motor deficit, despite the fact the initial severity can affect outcomes. Such information would be useful for clinicians to determine the treatment plan according to the severity of the motor deficit and follow changes in motor function.

The aim of this work was to describe the dose of robotic motor rehabilitation using the framework described by Hayward et al. [14] in a sample of people with upper limb hemiparesis in the subacute phase after stroke. We described the dose both in terms of initial motor severity and in terms of recovery: “good” and “poor” recoverers.

## 2. Materials and Methods

### 2.1. Participants

We conducted a retrospective study of data from a cohort of thirty-six people with hemiparesis (mean age 59 (16) years, 15 females; see all demographic and clinical characteristics in results section) who underwent an upper limb rehabilitation program combining robotic therapy and conventional occupational therapy in the subacute phase of stroke. This study was carried out in accordance with current French legislation, and data processing was carried out within the framework of reference methodology No. 004 (MR004) [15]. All precautions to preserve the safety of the personal data processed (confidentiality, integrity and availability) were rigorously respected.

### 2.2. Training Intervention

**Figure 1:**
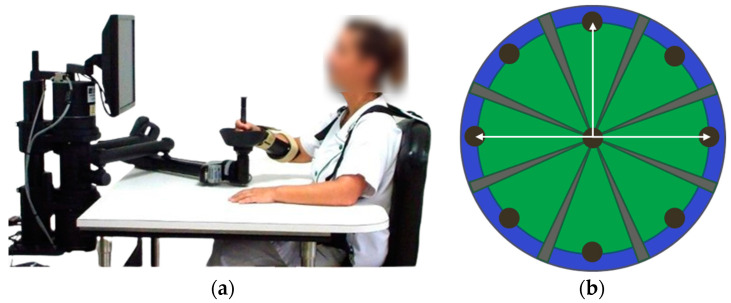
InMotion 2.0 shoulder/elbow robotic system. (**a**) Therapist using the InMotion 2.0 shoulder/elbow robotic system; (**b**) pointing task interface, the white arrows represent the trajectory directions used for kinematic analysis.

All participants underwent an upper limb rehabilitation program involving robotic therapy and occupational therapy. In conventional occupational therapy, interventions involved passive muscle stretches performed by a clinician, active reaching movements and specific grasping and releasing tasks performed by the participant. These exercises were aimed at improving motor function, coordination and muscle activation. The frequency and intensity of the sessions were tailored to each patient’s abilities and progression. In robotic therapy, participants performed many repeated movements with the paretic arm using an end-effector type robotic device (Figure 1a). This involved performing goal-directed shoulder and elbow movements in the horizontal plane while holding the handle of the robotic arm with the distal part of the upper limb. The circular pointing task consisted of repeated movements from a rest position (center) towards eight visual targets located around a circle (Figure 1b). The distance to travel between the center and each target could be chosen from three options (3, 9 or 14 cm, i.e., the maximum distance).

The combined robotic and occupational therapy was provided for 4 weeks, 4 to 5 days a week, with one 60 min session per day for each. The core structure of the robotic therapy protocol was the same for all participants; however, the difficulty of movements (i.e., the effort required by the participant to reach the targets) was adapted based on individual motor impairment. The physical modalities of the robotic therapy—modulating the level of effort required (assisted, unassisted or resisted)—were adjusted according to each patient’s abilities. This ensured an optimal challenge while maintaining a high number of repetitions. This personalized approach was designed to maximize motor engagement and enhance recovery potential.

### 2.3. Outcome Measures

All participants were assessed before and after the upper limb rehabilitation program using the FMA [16] for the upper limb and robot-derived kinematic parameters. The FMA quantifies motor deficiencies in the joint segments of the arm. This outcome measure is reliable, sensitive to change and has been validated for use in spastic paresis in the sub-acute phase of stroke [17,18]. The maximum score is 66 points, with higher scores indicating lower levels of impairment.

Kinematic analysis was performed on raw data measured and recorded by the robot during unassisted, planar movements from the center of the circle to one of eight equidistant targets placed 14 cm from the center. The trajectory data, consisting of *x* and *y* coordinates over time, were extracted for each movement and subsequently processed using MATLAB R2024b to compute the kinematic parameters. A total of 80 movements were performed. To optimize the analysis, only trajectories in three specific directions (front and side; Figure 1b), classically considered the most difficult for the paretic upper limb, were included in the calculation of kinematic parameters. Four parameters were computed (Table 1):

All kinematic parameters were normalized by values from healthy individuals and expressed as a percentage of those values.

### 2.4. Data Analysis and Statistical Analysis

The cohort was initially stratified by motor-severity-based categories using the stratification of Woodbury et al. [19]: severe (0 < FM score ≤ 19 pts), moderate (20 ≤ FM score ≤ 47 pts) and mild (FM score ≥ 48 pts) [19]. A change in motor severity category between the start and the end of the upper limb rehabilitation program separated the cohort into good and poor recoverers. This method was used to express a clinically important change in motor function. Dose articulation was described in robotic therapy using the framework of Hayward et al. (Table 2).

Normality tests were not conducted prior to applying *t*-tests and ANOVA, as these tests are known to be robust to deviations from normality. However, when appropriate, non-parametric alternatives such as the Wilcoxon test for paired data and the Mann–Whitney test for independent samples were used to ensure the robustness of our statistical analysis. To analyze the treatment effects on the clinical scores and on kinematic parameters, paired *t*-tests were used to compare pre- and post-treatment data following the combined rehabilitation program (N = 36), with Cohen’s d reported as a measure of effect size. Wilcoxon tests were used to compare pre- and post-intervention treatment effects across participants with severe, moderate and mild impairments, with r values reported as a measure of effect size. Finally, Mann–Whitney tests were used to compare poor vs. good recoverers between the pre- and post-intervention treatment effects with r values reported as a measure of effect size; in cases where the *p*-value was <0.05, Wilcoxon tests were used as post hoc tests.

Correlations between the initial motor impairment severity (FMA) and all pre-treatment kinematic outcomes were analyzed using Spearman’s rho coefficient. Correlations between the initial motor impairment severity and time spent in each robot physical modality were explored using Spearman’s rho coefficient.

A two-way repeated measures ANOVA was performed only on the severe impairment group, comparing poor and good recoverers, to assess differences in physical modality use over five robotic sessions (S1, S4, S8, S12 and S16), with partial eta squared reported as a measure of effect size. If the ANOVA result was significant, multiple comparisons with Bonferroni corrections between sessions were performed for each modality. For all statistical tests, significance was set to *p* < 0.05.

## 3. Results

### 3.1. Baseline Characteristics

Thirty-six individuals were included in this study, twenty-three of whom had severe initial impairment (mean (SD) initial FMA score of 12 (6) points), eight had moderate impairment (initial FMA score of 37 (8) points) and five had mild impairment (initial FMA score of 53 (2) points). In the severely impaired group, twelve were good recoverers with an initial FMA score of 15 (5) points (vs. 9 (6) points for poor recoverers (N = 11)). In the moderate impairment group, three participants were classed as good recoverers, with an initial FMA score of 41 (12) points (vs. 34 (3) points for poor recoverers (N = 5)). Participants’ baseline characteristics are presented in Table 3.

### 3.2. Clinical Outcomes

**Figure 2:**
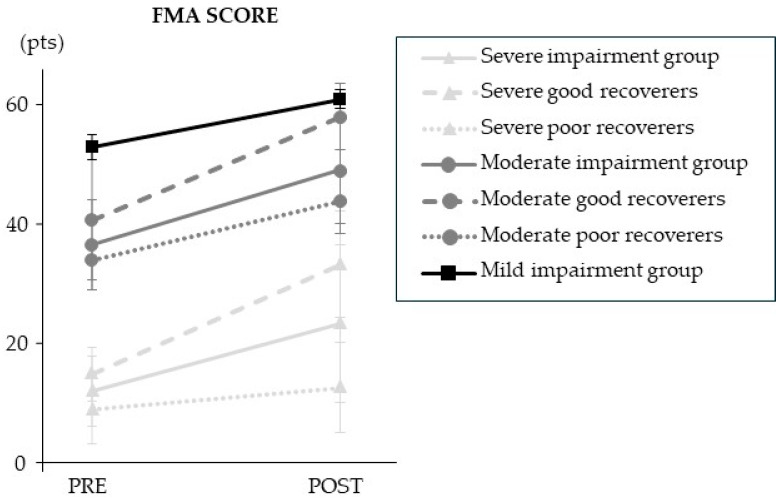
Change in FMA score from pre- to post-combined training. Data are mean (SD).

The clinical outcomes are presented in Figure 2 and Table 4. Overall, the results showed a statistically significant mean increase in total FMA score of 11 (10) points after the combined program (*p* = 4.12 × 10^−8^). The stratified results showed that FMA scores changed from pre- to post-intervention in participants with severe (+11 (12) points; *p* = 6.90 × 10^−5^) and moderate (+13 (6) points; *p* = 7.81 × 10^−3^) impairment. Improvement in poor and good recoverers only differed significantly in the severely impaired group (*p* = 4.92 × 10^−4^); poor recoverers improved by 4 (4) points and good recoverers improved by 18 (12) points. FMA score did not improve after the combined program in the mild impairment group (+8 (2) points; NS).

### 3.3. Kinematic Parameter Outcomes

**Figure 3:**
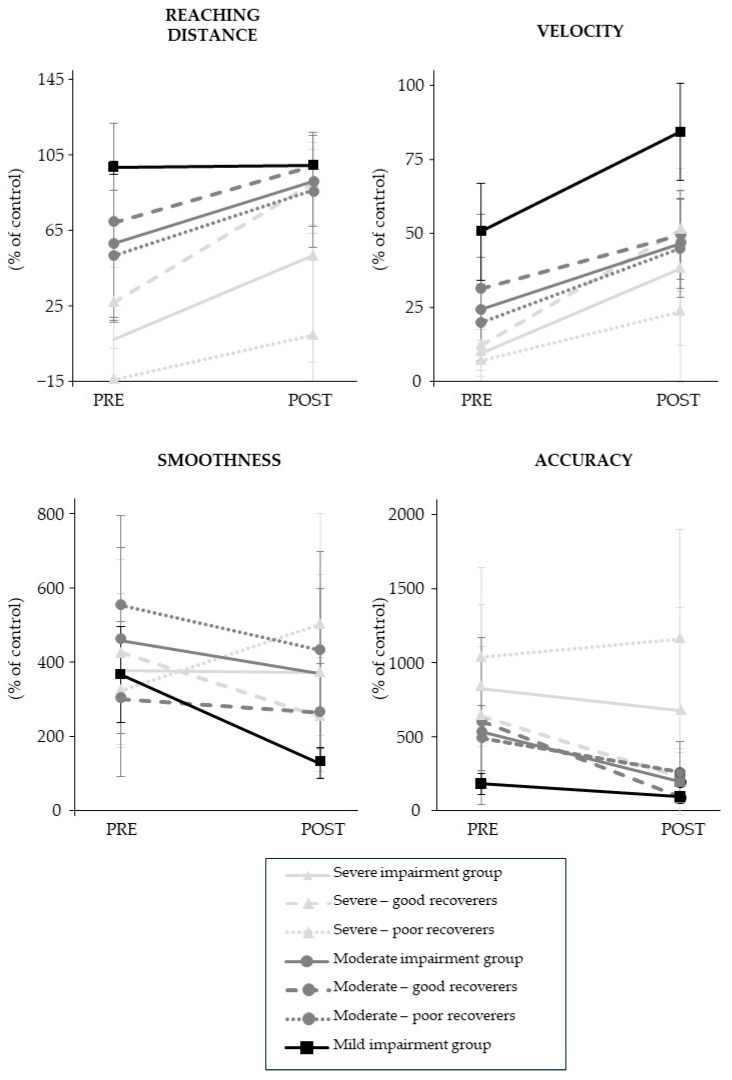
Robot-based kinematic outcomes. Data are mean (SD).

The kinematic outcomes are summarized in Figure 3. The results showed that the mean reaching distance (+36 (47) %; *p* = 5.02 × 10^−4^, Cohen’s d = 0.8) and velocity (+28 (22) %, *p* = 4.92 × 10^−4^, Cohen’s d = 1.3) improved at the end of the combined rehabilitation program.

The stratified results showed that reaching distance (+44 (51) %; *p* = 3.76 × 10^−4^, effect size = 0.5) and velocity (+28 (23) %, *p* = 6.09 × 10^−6^, effect size r = 0.6) improved in participants with severe impairment, and velocity (+22 (20) %; *p* = 1.53 × 10^−2^, effect size r = 0.5) and accuracy (−336 (360) %, *p* = 3.37 × 10^−2^, effect size r = 0.6) improved in those with moderate impairment. In the mild impairment group, velocity (34 (20) %, *p* = 1.80 × 10^−2^, effect size r = 0.6), accuracy (−89 (44) %, *p* = 1.08 × 10^−2^, effect size r = 0.6) and smoothness (−240 (119) %, *p* = 1.08 × 10^−2^, effect size r = 0.6) improved.

The difference between poor and good recoverers was only significant in the severe impairment group for velocity (*p* = 1.06 × 10^−2^, effect size r = 0.5) and smoothness (*p* = 5.09 × 10^−3^, effect size r = 0.6). In poor recoverers, velocity improved by 17 (20) % but smoothness deteriorated by 177 (293) %. In good recoverers, velocity improved by 39 (21) % and smoothness improved by −176 (270) %.

**Figure 4:**
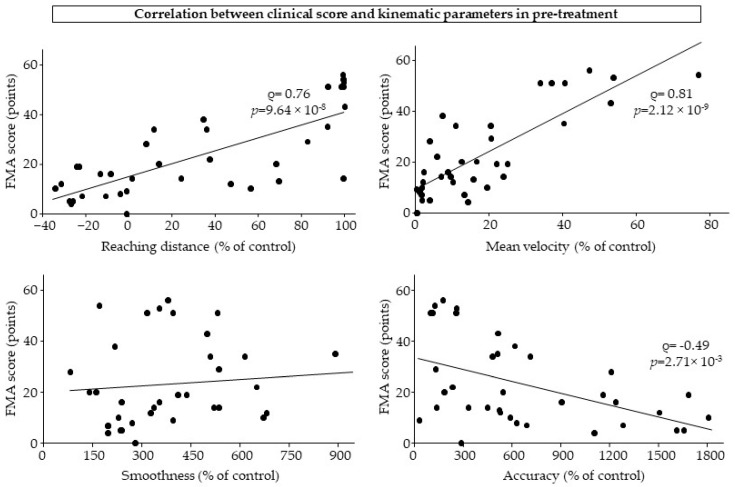
Pre-treatment correlation between FMA score and kinematic parameters.

The correlation results are shown in Figure 4. Pre-treatment, FMA scores were strongly correlated with reaching distance (Spearman’s rho = 0.76; *p* = 9.64 × 10^−8^) and mean velocity (Spearman’s rho = 0.81; *p* = 2.12 × 10^−9^) and moderately negatively correlated with accuracy (Spearman’s rho = −0.49; *p* = 2.71 × 10^−3^).

### 3.4. Dose of Robotic Training

The dose of robotic training described using Hayward’s framework is reported in Table 5. Overall, this description showed that severely impaired participants performed 76% of assisted movements, 17% of unassisted movements and 7% of resisted movements. Good recoverers performed 63% of assisted movements, 25% of unassisted movements and 12% of resisted movements whereas poor recoverers performed 94% of assisted movements.

The use of robot modalities was similar between participants with moderate impairment and good recoverers with severe impairment: 58% of movements were assisted, 26% of movements were unassisted and 17% of movements were resisted. The use of each modality was relatively evenly distributed for the good recoverers, with 43% of assisted movements, 26% of unassisted movements and 30% of resisted movements. The use of each modality for the poor recoverers was similar to that of the good recoverers with severe impairment (i.e., 64% of movements were assisted, 26% of movements were assisted and 9% of movements were resisted).

Participants with mild impairment were more likely to use the unassisted modality (40% of movements) and the resisted modality (39%), and the assisted modality was only used for 18% of movements.

**Figure 5:**
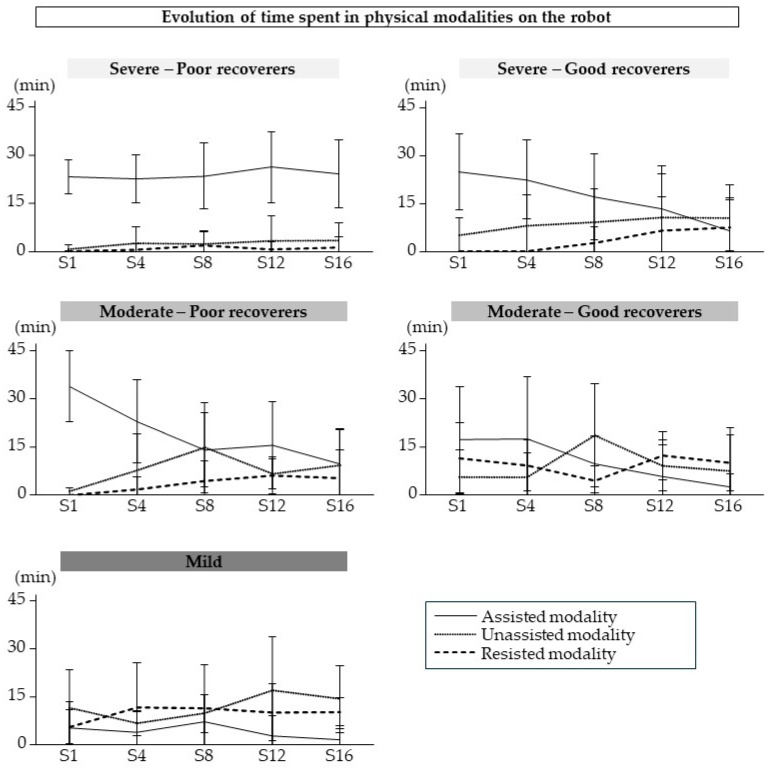
Change in use of each robot modality over time. Data are mean (SD).

The patterns of use of the physical modalities during training sessions with the robot are shown in Figure 5. The two-way repeated measures ANOVA indicated a significant modality x session interaction only in good recoverers with severe impairment (*p* = 1.56 × 10^−6^, η*_p_*^2^ = 0.4). The time spent in the assisted modality decreased over time for these participants (Session 1 vs. Session 16, *p* = 2.11 × 10^−4^; Session 4 vs. Session 12, *p* = 2.98 × 10^−3^; Session 4 vs. Session 16, *p* = 2.23 × 10^−4^).

**Figure 6:**
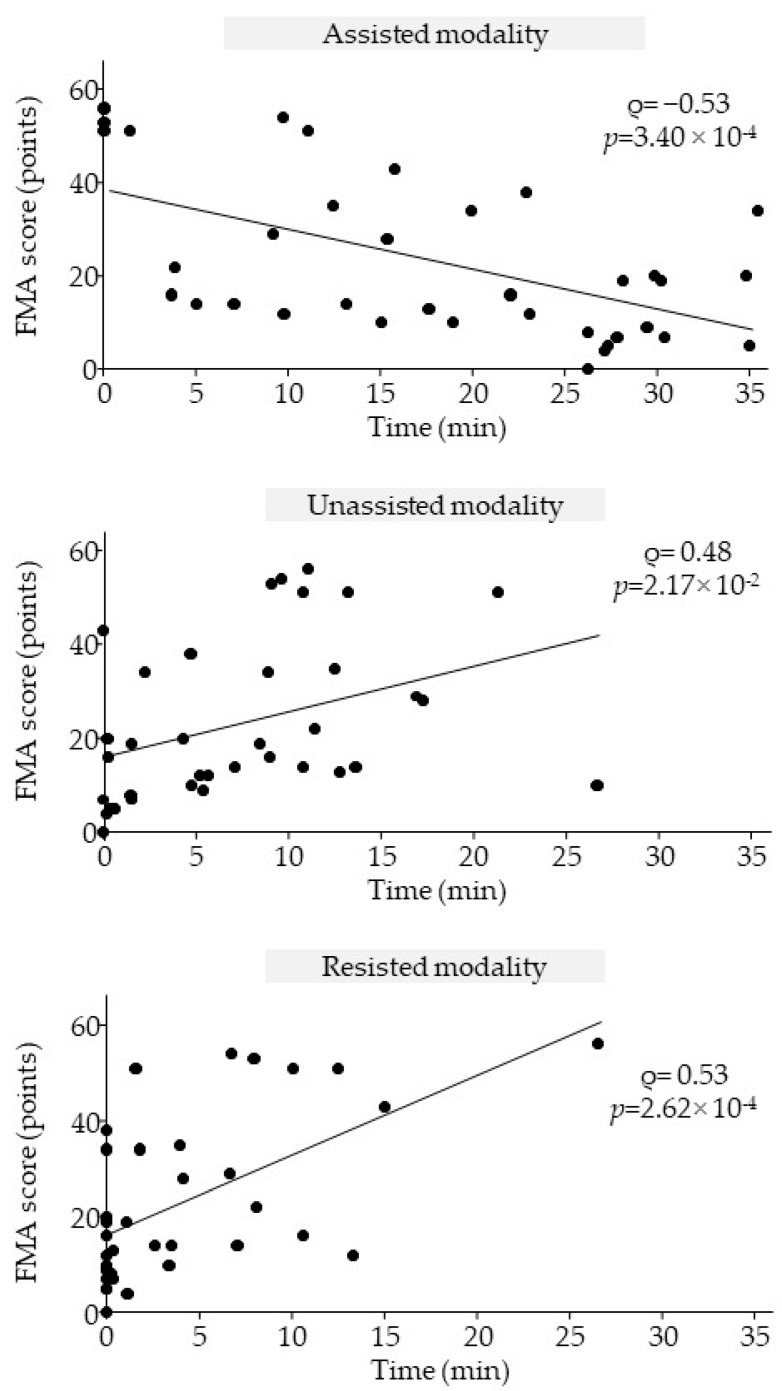
Correlation between time spent in each modality and Fugl-Meyer Assessment (FMA) score at pre-treatment.

The correlation results are shown in Figure 6. Initial FMA scores were moderately negatively correlated with mean time spent in assisted modality (Spearman’s rho = −0.53; *p* = 3.40 × 10^−4^) and moderately correlated with mean time spent in unassisted modality (Spearman’s rho = 0.48; *p* = 2.17 × 10^−2^) and with mean time spent in resisted modality (Spearman’s rho = 0.53; *p* = 2.62 × 10^−4^).

## 4. Discussion

The aim of this retrospective study was to accurately describe and monitor the dose of upper limb motor training using a robotic device administered to a cohort of people after stroke with subacute paresis. The robotic training was combined with conventional upper limb care. The originality of this work lies in the double stratification: (1) the initial severity of the motor deficit, and (2) a change in motor severity category, which defined good and poor recoverers.

The results of this study showed that clinical scores improved in all participants, regardless of the initial motor deficit level. However, the sub-cohort of participants with initial severe impairment clearly showed two recovery profiles despite the similar initial deficit. FMA score improved by 18 points in good recoverers after the combined program, whereas it only improved by 4 points in poor recoverers. The main finding of this study indicates that the use of physical modalities is dependent on the level of initial motor impairment but may also be a critical determinant of motor outcomes.

Participants with the most severe impairment, particularly those with FMA < 10 pts, predominantly used the assisted modality to perform repeated movements, which may be insufficiently challenging to promote cortical stimulation [20]; conversely, those with less severe impairment used unassisted and resisted modalities.

### 4.1. Relevance of Parameters to Monitor Paretic Upper Limb Training After Stroke

In the literature, the dose of upper limb rehabilitation is often measured by the scheduled duration of therapy, a variable considered relevant for quantifying physical training [21]. The results of a meta-analysis showed a positive relationship between the scheduled duration of therapy and motor outcomes; thus, higher doses of therapy could lead to clinically significant improvements [6]. In the present study, all participants received 60 min of scheduled robotic therapy, but the associated motor outcomes differed between groups and subgroups, showing that this parameter is not sufficient to describe the dose and then interpret the results according to changes in motor impairment. The parameter of time spent in active intervention seems more relevant, as it objectively quantifies the number of minutes the participant is active on the task; this study showed that participants were only active for 57% of the scheduled session time (60 min), which is far shorter than the scheduled time in actual practice. These results are consistent with the literature [22,23], but again, this parameter alone cannot describe practice dosage [24,25] since the amount of rehabilitation time was the same for all participants.

Similarly, counting the number of movement repetitions does not describe the dose of practice. This parameter is interesting, but its use independently of the effort associated with the movement is insufficient to quantify practice intensity [26].

Hayward et al. [14] dissected each motor task performance episode into the combined description of duration (in minutes), intensity (repetitions/second) and difficulty (physical effort). Using this methodological framework, the results of the present study showed that the cohort performed 457 assisted movements (53% of total active time), 174 unassisted movements (22% of total active time) and 118 movements against resistance (12% of total active time). This more detailed description provides an accurate representation of what people do during robotic therapy. Dose quantification can also stimulate the individual by providing feedback on motor performance and managing the difficulty of the movements performed. Our results reveal significant differences in the use of robotic therapy modalities, both across motor severity levels and recovery patterns. Specifically, we observed that as motor deficits become less severe, the use of the assisted modality decreases. In contrast, both the unassisted and resisted modalities are used more frequently as motor function improves, with a more pronounced increase in the use of the resisted modality. This trend suggests an evolution in the use of physical modalities during rehabilitation, which has been relatively underexplored in the existing literature.

In a similar vein, Stein et al. [27] progressively integrated the resisted modality into their robotic therapy protocol. However, their study suggested that resistance training did not significantly improve strength. This lack of improvement may have been due to suboptimal training stimuli, particularly in terms of repetition numbers (which were not reported). Consequently, comparing our results with the existing literature on this topic is challenging due to variations in training protocols and the absence of detailed repetition data in previous studies.

Additionally, we observed notable differences in modality usage between good and poor recoverers. In both severe and moderate impairments, poor recoverers predominantly relied on assisted modality. In contrast, good recoverers not only used assistance but also engaged in unassisted movements (for severe and moderate impairments) and resistance training (for moderate impairments only). These findings suggest a progression towards greater autonomy in rehabilitation, reflecting a tailored adaptation of task difficulty by therapists based on initial motor performance and recovery progression. This adaptation supports the notion that therapeutic interventions can be individualized to optimize patient outcomes.

### 4.2. Tailoring Exercise Difficulty: The Impact of Manipulating Physical Treatment Modalities in Robotic Therapy

In this study, the difficulty of motor training was related to the physical effort requirement and probably to the participant’s intention to be engaged in performing repeated movements. In robotic rehabilitation, this difficulty is modulated by manipulating the training modalities and the physical interactions between the user and the robotic arm. In this form of physical therapy, the effort required for each physical modality can be ranked in an orderly fashion; a movement against resistance will require greater effort than an unassisted movement, which in turn will require greater effort than an assisted movement. It is therefore not surprising to observe in this study that the participants with more severe motor deficits spent more time performing assisted than unassisted or resisted movements. Conversely, those with milder motor deficits spent more time performing resisted movements.

It is difficult to compare this observation with the literature as few studies have investigated the change in time spent using each modality over time as a function of the initial motor deficit. According to the literature, the assisted modality is predominantly used [13]. In this study, the participants—whether mild, moderate or severe—were active for the same amount of time (34 min on average) and performed a comparable number of movements, averaging between 600 and 900 movements per session. However, describing these parameters in terms of the modality used demonstrates how therapists managed the effort demand on the participants.

### 4.3. Identification of Physical Modality Use Patterns

The results of this study demonstrated that there are four therapeutic profiles in the use of robotic modalities depending on the evolution of motor performance over the course of the program. In order of difficulty, they are as follows:

Profile A: Use of assisted modality (A)

Profile B: Integration of unassisted modality (A + B)

Profile C: Integration of the resisted modality (B + C)

Profile D: Leaving assisted modality (B − A + C)

For the participants with very severe impairments (FMA < 10 pts), the therapist decided to use the assisted modality for the duration of the program (profile A). Those whose motor performance was deemed sufficient by the therapist were able to benefit from the unassisted modality in addition to the assisted modality (i.e., simultaneous use of assisted and unassisted modalities; profile B). For participants with the most favorable evolution pattern, the therapist decided to integrate the third physical modality available on the robot, the resisted modality (profile C). Finally, a last therapeutic profile appears for a small proportion of participants for whom the therapist has decided to stop using the assisted modality and use only two physical modalities, unassisted and resisted (profile D).

### 4.4. Therapist’s Role in Adjusting Robotic Therapy Training Parameters for Individuals with the Most Severe Impairment

Although robotic therapy seems appropriate for people with minimal upper-limb motor function, the literature highlights the potential slacking effect (i.e., individuals allow themselves to be carried along by the assistance, with minimal participation [28,29]. The results of the present study showed that a subgroup of individuals with severe and moderate impairment can generate sufficient motor activity on the robot to achieve a favorable outcome. Analysis of the physical training modalities used by these subgroups enables them to be identified at mid-treatment (S8); these participants benefited from all physical modalities at this stage, with partial release from assistance. This result is consistent with one of the fundamental principles governing motor learning after stroke, “use it and improve it or lose it” [30].

**Figure 7:**
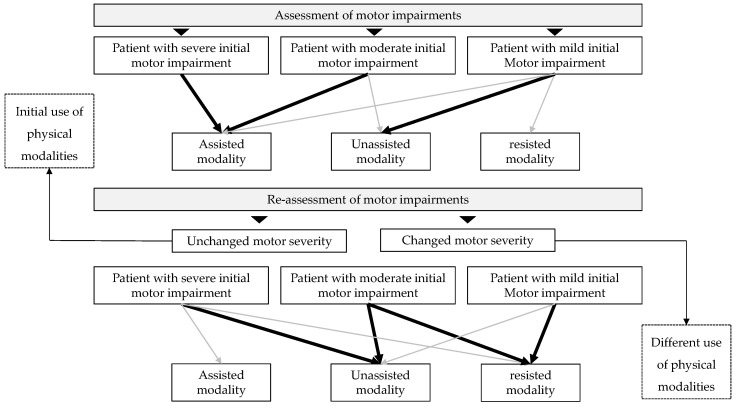
Decision tree for tailoring physical modalities in robotic therapy. In the decision tree, thick black arrows indicate high usage, while thin gray arrows indicate low usage.

In individuals with severe and moderate motor impairments, the use of robotic assistance should be as short as possible to encourage the most active behavior possible. Robotic algorithms are programmed to adapt to the individual’s motor behavior and, therefore, to their engagement level in the task and not actually to their motor capacity. If the robot performs the movement for the individual, or conversely, if the individual performs the movement with assistance too easily, they will perform many movements without generating effort. In both situations, the therapist’s supervision is critical to adapt the program to the individual’s needs, even during the session if fatigue occurs. The therapist can also use performance and assistance indicators to adjust the difficulty, either modifying the exercise parameters or proposing a higher-level physical modality, such as the unassisted or resisted modality. A study showed the importance of considering the severity of upper-limb paresis when deciding on the level of assistance [31]. The authors suggested increasing the level of robotic assistance for individuals with severe to moderate paresis (FMA < 30) and reducing assistance for those with mild paresis. The results of the present study are consistent with these recommendations. However, it is strongly recommended that assistance be used for as short a time as possible to promote repetition of unassisted movements [20]. A decision tree has been developed to help therapists adjust the physical modalities of the robotic therapy (Figure 7).

### 4.5. Is Robotic Therapy Inappropriate for People with Very Severe Impairment?

Considering the progression of kinematic parameters in “poor recoverers”, the changes were discrete, with improvements in range and speed of active movement. This suggests that, despite the use of assistance, the movements produced by these participants were at least partially active. These individuals likely need more prolonged therapy to achieve more significant motor outcomes. Furthermore, analysis of kinematic parameters showed different improvement strategies depending on the initial severity of the motor deficit. At the end of the combined program, those with severe impairment could use the robot to reach targets faster and further—a more quantitative strategy; those with moderate impairment were also able to reach targets faster but more accurately; and those with mild impairment could reach targets faster, more accurately and more smoothly—a more qualitative strategy. In the severe impairment group, the significant increase in reaching distance and velocity suggests an improvement in the ability to initiate and execute reaching movements, which is a fundamental component of functional arm use in daily activities. In the moderate impairment group, the improvements in velocity and accuracy indicate better movement efficiency and control, which may translate into improved precision in tasks such as grasping or object manipulation. In the mild impairment group, the improvements in velocity, accuracy, and smoothness suggest a more refined and coordinated motor execution, which is critical for fine motor tasks. Additionally, the analysis comparing poor and good recoverers revealed significant differences in velocity and smoothness in the severe impairment group, highlighting that individuals with better recovery potential demonstrated greater improvements in movement smoothness. This finding suggests that smoothness could be an important marker of motor recovery and may have implications for individualized rehabilitation strategies. Using this analysis to complement clinical analysis provides more insight into recovery processes and can help define which therapy should be administered to whom and when. Although the relevance of kinematic analysis seems obvious, the question of its true significance has yet to be answered. It is unclear whether improvements in kinematic parameters represent true recovery or an adaptation to the task.

### 4.6. Limitations

This retrospective study has several limitations. The sample size was relatively small, particularly for the subgroups with moderate and mild motor impairments, limiting the generalizability of the findings. The retrospective nature of this study also limits the generalizability of the results and the ability to control confounding factors as effectively as in a randomized controlled trial. However, to mitigate this limitation, subgroup analyses based on initial motor severity were performed. This study focused on a limited set of kinematic parameters, potentially neglecting other important aspects of motor recovery, such as muscle activation patterns and cortical activation. Furthermore, the kinematic parameter assessment involved a similar motor task to that used in rehabilitation; thus, improvement may also reflect a task-learning effect rather than a true improvement in motor function. Neuroradiological and/or neurophysiological evaluation of the cortico-spinal tract would help to explore underlying lesional profiles in people with severe impairment. In addition, this study did not investigate the neural mechanisms of motor recovery, such as changes in brain structure and function, or the relationship between these changes and the effects of robotic therapy. Future research integrating neuroimaging and neurophysiological techniques could provide deeper insights into the neural plasticity promoted by robotic therapy.

## 5. Conclusions

This study highlights the importance of tailoring robotic therapy to the individual needs of people after a stroke. By stratifying individuals according to their initial motor severity and motor outcomes, we can identify distinct recovery patterns that could help optimize treatment strategies. A multidimensional approach to quantifying dose, considering factors such as physical modality, intensity and duration, provides valuable insights into the treatment dose-related motor outcome. Although robotic therapy shows promise for improving motor function, future research should explore its long-term effects and investigate the neural mechanisms underlying recovery. Larger, prospective studies are needed to validate these findings and refine clinical practice.

## Figures and Tables

**Table 1:** Kinematic parameters.

Parameter	Definition	Interpretation
Mean velocity (cm/s)	Average speed during movement from center to target	Higher values indicate better motor efficiency
Smoothness (number of velocity peaks)	Number of peaks in the velocity profile	Fewer peaks suggest smoother movement
Reaching distance (cm)	Distance between target center and the orthogonal projection of final hand position (cm)	Higher values indicate greater distance covered
Path Accuracy (cm)	Root mean square error (deviation) from the straight line	Lower values indicate higher accuracy

**Table 2:** Dose articulation framework based on Hayward et al. [14].

Frame of Reference	Item	Dose Dimension
Intervention	1	Duration (week)
	2	Days (day/week)
	3	Sessions (session/day)
Session	4	Session length (min)
	5	Session density: active and inactive (min)
Episode (s)	6	Length (min)
	7	Difficulty (low/medium/high)
	8	Intensity (total movement repetitions)

**Table 3:** Participants’ baseline characteristics.

		N	Mean Age (SD) [Years]	Sex [M/F]	Side of Paresis [R/L]	Type of Stroke [I/H]	Mean Time Post-Stroke (SD) [Days]	Initial FMA Score (SD) [/66 pts]
All participants		36	59 (16)	21/15	19/17	25/11	54 (26)	23 (17)
Severely impaired		23/36	56 (18)	12/11	14/9	16/7	57 (27)	12 (6)
	Poor recoverers	11/23	57 (19)	5/6	6/5	9/2	67 (33)	9 (6)
	Good recoverers	12/23	55 (18)	7/5	8/4	7/5	49 (19)	15 (5)
Moderately impaired		8/36	64 (11)	5/8	3/5	6/2	59 (26)	37 (8)
	Poor recoverers	5/8	64 (13)	3/2	2/3	4/1	64 (23)	34 (3)
	Good recoverers	3/8	66 (10)	2/1	1/2	2/1	51 (33)	41 (12)
Mildly impaired		5/36	61 (9)	4/1	2/3	3/2	33 (9)	53 (2)

**Table 4:** Description of changes in FMA score pre- to post-intervention. Data are mean (SD). N/A, not applicable.

		Mean FMA Score Before Training (SD) [pts]	Mean FMA Score After Training (SD) [pts]	Mean Change in FMA Score After Training (SD) [pts]	*p*-Values	Effect Size
All participants		23 (17)	34 (19)	11 (10)	4.12 × 10^−8^	1.2
Severely impaired	all	12 (6)	23 (13)	11 (12)	6.90 × 10^−5^	0.6
Poor recoverers		9 (6)	13 (8)	4 (4)	1.64 × 10^−2^	0.5
Good recoverers		15 (5)	33 (9)	18 (12)	2.49 × 10^−3^	0.6
Moderately impaired	all	37 (8)	49 (9)	12 (6)	7.81 × 10^−3^	0.6
Poor recoverers		34 (3)	44 (5)	10 (4)	0.06	N/A
Good recoverers		41 (12)	58 (6)	17 (7)	0.25	N/A
Mildly impaired	all	53 (2)	61 (2)	8 (2)	0.06	N/A

**Table 5:** Dose results for robot training. Data are mean (SD).

		All Participants	Severely Impaired			Moderately Impaired			Mildly Impaired
			All	Poor Recoverers	Good Recoverers	All	Poor Recoverers	Good Recoverers	All
	N	36	23	11	12	8	5	3	5
Intervention	Duration (week)	4.9 (1.6)	4.9 (1.5)	5.4 (1.8)	4.5 (1.1)	5.6 (2.1)	5.8 (2.7)	5.4 (0.4)	3.9 (0.8)
	Days (day/week)	3.6 (1.0)	3.6 (1.0)	3.4 (1.1)	3.8 (0.9)	3.2 (0.8)	3.3 (1.0)	3.0 (0.2)	4.4 (0.9)
	Sessions (s/day)	1							
Session	Length (min)	60							
	Active (min)	34.3 (8.3)	33.5 (5.6)	33.0 (4.7)	34.0 (6.4)	36.7 (8.7)	35.9 (4.7)	37.8 (5.8)	33.5 (5.7)
	Inactive (min)	25.7 (8.3)	26.5 (5.6)	27.0 (4.7)	26.0 (6.4)	23.3 (8.7)	24.1 (4.7)	22.2 (5.8)	26.5 (5.7)
Episode 1	Length (min)	3.9 (4.6)	3.7 (2.9)	3.3 (3.2)	4.1 (2.7)	3.8 (4.4)	2.6 (1.0)	7.2 (1.3)	3.7 (2.3)
	Difficulty	Game. active							
	Intensity (rep/s)	NA							
Episode 2	Length (min)	18.0 (13.4)	21.4 (10.1)	26.0 (7.2)	17.2 (10.7)	16.7 (13.4)	20.0 (10.3)	10.4 (9.0)	4.4 (5.5)
	Difficulty	Assisted. pointing task							
	Intensity (rep/s)	457 (356)	523 (296)	587 (270)	465 (318)	416 (310)	467 (179)	323 (287)	196 (260)
Episode 3	Length (min)	7.4 (9.5)	5.7 (6.4)	2.6 (4.2)	8.5 (6.9)	9.3 (10.8)	9.0 (5.9)	10.2 (9.0)	12.3 (5.1)
	Difficulty	Unassisted. pointing task							
	Intensity (rep/s)	174 (268)	114 (137)	37 (66)	183 (149)	189 (243)	189 (168)	197 (196)	442 (303)
Episode 4	Length (min)	4.1 (7.6)	2.2 (3.8)	0.5 (1.1)	3.8 (4.8)	5.1 (7.0)	2.5 (2.8)	9.7 (5.4)	11.0 (9.5)
	Difficulty	Resisted. pointing task							
	Intensity (rep/s)	118 (231)	50 (89)	8 (14)	88 (111)	123 (165)	65 (75)	227 (130)	416 (333)

## Data Availability

All data are available in electronic format at the Centre de Réadaptation Fonctionnelle (CRF) Les Trois Soleils.

## Abbreviations

The following abbreviations are used in this manuscript:

FMA	Fugl-Meyer Assessement

## References

[1] Lawrence E.S., Coshall C., Dundas R., Stewart J., Rudd A.G., Howard R., Wolfe C.D.A. (2001). Estimates of the Prevalence of Acute Stroke Impairments and Disability in a Multiethnic Population. Stroke.

[2] Puig J., Blasco G., Schlaug G., Stinear C.M., Daunis-i-Estadella P., Biarnes C., Figueras J., Serena J., Hernández-Pérez M., Alberich-Bayarri A. (2017). Diffusion Tensor Imaging as a Prognostic Biomarker for Motor Recovery and Rehabilitation after Stroke. Neuroradiology.

[3] Prabhakaran S., Zarahn E., Riley C., Speizer A., Chong J.Y., Lazar R.M., Marshall R.S., Krakauer J.W. (2008). Inter-Individual Variability in the Capacity for Motor Recovery after Ischemic Stroke. Neurorehabilit. Neural Repair.

[4] Kwakkel G., Stinear C., Essers B., Munoz-Novoa M., Branscheidt M., Cabanas-Valdés R., Lakičević S., Lampropoulou S., Luft A.R., Marque P. (2023). Motor Rehabilitation after Stroke: European Stroke Organisation (ESO) Consensus-Based Definition and Guiding Framework. Eur. Stroke J..

[5] Kwakkel G., Wagenaar R.C., Twisk J.W.R., Lankhorst G.J., Koetsier J.C. (1999). Intensity of Leg and Arm Training after Primary Middle-Cerebral-Artery Stroke: A Randomised Trial. Lancet.

[6] Kwakkel G., Van Peppen R., Wagenaar R.C., Dauphinee S.W., Richards C., Ashburn A., Miller K., Lincoln N., Partridge C., Wellwood I. (2004). Effects of Augmented Exercise Therapy Time After Stroke: A Meta-Analysis. Stroke.

[7] Bütefisch C., Hummelsheim H., Denzler P., Mauritz K.H. (1995). Repetitive Training of Isolated Movements Improves the Outcome of Motor Rehabilitation of the Centrally Paretic Hand. J. Neurol. Sci..

[8] Feys H., De Weerdt W., Verbeke G., Steck G.C., Capiau C., Kiekens C., Dejaeger E., Van Hoydonck G., Vermeersch G., Cras P. (2004). Early and Repetitive Stimulation of the Arm Can Substantially Improve the Long-Term Outcome after Stroke: A 5-Year Follow-up Study of a Randomized Trial. Stroke.

[9] Lum P.S., Burgar C.G., Shor P.C., Majmundar M., Van der Loos M. (2002). Robot-Assisted Movement Training Compared with Conventional Therapy Techniques for the Rehabilitation of Upper-Limb Motor Function after Stroke. Arch. Phys. Med. Rehabil..

[10] Masiero S., Armani M., Ferlini G., Rosati G., Rossi A. (2014). Randomized Trial of a Robotic Assistive Device for the Upper Extremity during Early Inpatient Stroke Rehabilitation. Neurorehabil. Neural Repair.

[11] Volpe B.T., Krebs H.I., Hogan N., Edelstein L., Diels C., Aisen M. (2000). A Novel Approach to Stroke Rehabilitation: Robot-Aided Sensorimotor Stimulation. Neurology.

[12] Lo A.C., Guarino P.D., Richards L.G., Haselkorn J.K., Wittenberg G.F., Federman D.G., Ringer R.J., Wagner T.H., Krebs H.I., Volpe B.T. (2010). Robot-Assisted Therapy for Long-Term Upper-Limb Impairment after Stroke. N. Engl. J. Med..

[13] Basteris A., Nijenhuis S.M., Stienen A.H.A., Buurke J.H., Prange G.B. (2014). Training Modalities in Robot-Mediated Upper Limb Rehabilitation in Stroke: A Framework for Classification Based on a Systematic Review. J. Neuroeng. Rehabil..

[14] Hayward K.S., Churilov L., Dalton E.J., Brodtmann A., Campbell B.C.V., Copland D., Dancause N., Godecke E., Hoffmann T.C., Lannin N.A. (2021). Advancing Stroke Recovery Through Improved Articulation of Nonpharmacological Intervention Dose. Stroke.

[15] Gorphe P., Jannin C. (2019). Regulatory Aspects of Prospective and Retrospective Clinical Research in France in 2018. Eur. Ann. Otorhinolaryngol. Head Neck Dis..

[16] Gladstone D.J., Danells C.J., Black S.E. (2002). The Fugl-Meyer Assessment of Motor Recovery after Stroke: A Critical Review of Its Measurement Properties. Neurorehabil. Neural Repair.

[17] Sanford J., Moreland J., Swanson L.R., Stratford P.W., Gowland C. (1993). Reliability of the Fugl-Meyer Assessment for Testing Motor Performance in Patients Following Stroke. Phys. Ther..

[18] Duncan P.W., Propst M., Nelson S.G. (1983). Reliability of the Fugl-Meyer Assessment of Sensorimotor Recovery Following Cerebrovascular Accident. Phys. Ther..

[19] Woodbury M.L., Velozo C.A., Richards L.G., Duncan P.W. (2013). Rasch Analysis Staging Methodology to Classify Upper Extremity Movement Impairment after Stroke. Arch. Phys. Med. Rehabil..

[20] Bo Nielsen J., Willerslev-Olsen M., Christiansen L., Lundbye-Jensen J., Lorentzen J. (2015). Science-Based Neurorehabilitation: Recommendations for Neurorehabilitation from Basic Science. J. Mot. Behav..

[21] Lohse K.R., Lang C.E., Boyd L.A. (2014). Is More Better? Using Metadata to Explore Dose−Response Relationships in Stroke Rehabilitation. Stroke.

[22] James J., Mcglinchey M.P. (2021). How Active Are Stroke Patients in Physiotherapy Sessions and Is This Associated with Stroke Severity?. Disabil. Rehabil..

[23] English C., Hillier S., Kaur G., Hundertmark L. (2014). People with Stroke Spend More Time in Active Task Practice, but Similar Time in Walking Practice, When Physiotherapy Rehabilitation Is Provided in Circuit Classes Compared to Individual Therapy Sessions: An Observational Study. J. Physiother..

[24] Kaur G., English C., Hillier S. (2012). How Physically Active Are People with Stroke in Physiotherapy Sessions Aimed at Improving Motor Function? A Systematic Review. Stroke Res. Treat..

[25] Clark B., Whitall J., Kwakkel G., Mehrholz J., Ewings S., Burridge J. (2021). The Effect of Time Spent in Rehabilitation on Activity Limitation and Impairment after Stroke. Cochrane Database Syst. Rev..

[26] Scrivener K., Sherrington C., Schurr K. (2012). Exercise Dose and Mobility Outcome in a Comprehensive Stroke Unit: Description and Prediction from a Prospective Cohort Study. J. Rehabil. Med..

[27] Stein J., Krebs H.I., Frontera W.R., Fasoli S.E., Hughes R., Hogan N. (2004). Comparison of Two Techniques of Robot-Aided Upper Limb Exercise Training after Stroke. Am. J. Phys. Med. Rehabil..

[28] Emken J.L., Bobrow J.E., Reinkensmeyer D.J. Robotic Movement Training as an Optimization Problem: Designing a Controller That Assists Only as Needed. Proceedings of the 9th International Conference on Rehabilitation Robotics (ICORR).

[29] Marchal-Crespo L., Reinkensmeyer D.J. (2009). Review of Control Strategies for Robotic Movement Training after Neurologic Injury. J. Neuroeng. Rehabil..

[30] Kleim J.A., Jones T.A. (2008). Principles of Experience-Dependent Neural Plasticity: Implications for Rehabilitation after Brain Damage. J. Speech Lang. Hear. Res..

[31] Takebayashi T., Takahashi K., Okita Y., Kubo H., Hachisuka K., Domen K. (2022). Impact of the Robotic-Assistance Level on Upper Extremity Function in Stroke Patients Receiving Adjunct Robotic Rehabilitation: Sub-Analysis of a Randomized Clinical Trial. J. Neuroeng. Rehabil..

